# Clinical Neuropathology practice news 2-2014: ATRX, a new candidate biomarker in gliomas 

**DOI:** 10.5414/NP300758

**Published:** 2014-03-26

**Authors:** Christine Haberler, Adelheid Wöhrer

**Affiliations:** Institute of Neurology, Medical University of Vienna, Austria

**Keywords:** gliomas, genomic aberrations, molecular biomarker, ATRX, IDH, 1p/19q

## Abstract

Genome-wide molecular approaches have substantially elucidated molecular alterations and pathways involved in the oncogenesis of brain tumors. In gliomas, several molecular biomarkers including *IDH* mutation, 1p/19q co-deletion, and MGMT promotor methylation status have been introduced into neuropathological practice. Recently, mutations of the *ATRX* gene have been found in various subtypes and grades of gliomas and were shown to refine the prognosis of malignant gliomas in combination with *IDH* and 1p/19q status. Mutations of *ATRX* are associated with loss of nuclear ATRX protein expression, detectable by a commercially available antibody, thus turning *ATRX* into a promising prognostic candidate biomarker in the routine neuropathological setting.

## Molecular biomarkers in gliomas 

In the last years, genome- and transcriptome-wide molecular approaches have deciphered molecular alterations and pathways involved in the oncogenesis of various brain tumors, especially in medulloblastomas and gliomas. This led to the introduction of molecular diagnostic, prognostic, and predictive biomarkers in neuro-oncology practice (for review see [1]). In gliomas, *IDH1* mutation, 1p/19q co-deletion, and *MGMT* promoter methylation can be routinely assessed and the results of these analyses help to refine the diagnosis and prognosis, and improve the treatment of the affected patients (for review see [2]). 

Recently, mutations in the *ATRX* (α-thalassemia/mental retardation syndrome X-linked) gene have been detected in gliomas of various subtypes and grades [3, 4, 5, 6, 7, 8, 9, 10]. In independent studies, a prognostic impact of *ATRX* mutations in the context of other molecular markers could be demonstrated [4, 11], thus turning *ATRX* into a new candidate biomarker for routine clinical practice. 

### ATRX 

The *ATRX* gene is located on chromosome Xq21.1 and encodes a protein that belongs to the H3.3-ATRX-DAXX chromatin-remodeling pathway. 

Mutations in *ATRX* give rise to characteristic developmental abnormalities including severe mental retardation, facial dysmorphism, urogenital abnormalities and α-thalassemia. *ATRX* is required for the incorporation of the histone variant H3.3 at pericentric heterochromatin and at telomeres, as well as at several transcription factor binding sites [12]. Perturbation of *ATRX* has been associated with a wide range of effects: altered patterns of DNA methylation (at subtelomeres, heterochromatic repeats, and ribosomal DNA), aberrant chromosome congression in mitosis, and segregation in meiosis, as well as telomere dysfunction [13]. 

### ATRX mutations in gliomas 

In 2011, mutations in the *ATRX* gene were described for the first time in a small fraction of adult and pediatric glioblastomas (GBM), as well as oligodendrogliomas (OG), and a significant correlation with alternative lengthening of telomeres (ALT), a presumed precursor to genomic instability, was demonstrated [3]. In 2012, mutations in the *H3F3A* and *ATRX* genes were detected in 30 – 40% of pediatric glioblastomas and in a smaller percentage of adult glioblastomas [9]. Analyses of *ATRX* in a series of 363 gliomas of various subtypes and grades revealed mutations in 25.6% of all tumors [4]. They occurred frequently in grade II astrocytomas (67%), grade III astrocytomas (73%), secondary GBMs (57%), and in tumors of mixed astrocytic and oligodendrocytic lineage (68%), whereas they were rare in primary GBMs (4%) and uncommon in pediatric GBMs (20%) and pure oligodendroglial tumors (14%). Furthermore, the correlation between *ATRX*, *p53*, and *IDH1/2* mutations, the status of chromosome arms 1p and 19q, as well as mutations of the *CIC* and *FUBP1* genes (candidate genes in 1p/19q co-deleted tumors) revealed a significant correlation between *ATRX*, *IDH1*, and *p53* mutations [4]. In contrast, *ATRX* mutation and combined 1p/19q co-deletion rarely co-occurred. Thus, based on the combination of these molecular changes, the authors defined three different groups of gliomas. 1) “group I-CF”(I*DH*-C*IC/*F*UBP1*): tumors with *IDH1/2* mutation and 1p/19q codeletion, *CIC* or *FUBP1* mutation, 2) “group I-A”: tumors with *IDH1/2* and *ATRX* mutation, 3) “group I-X”: tumors that did not fall into group 1 or 2. Correlations of these three groups with morphology, patient age and outcome revealed that astrocytic gliomas primarily had a I-A (32%) or I-X (66%) genetic signature, whereas oligodendroglial tumors were primarily I-CF (77%), but a significant subset harbored the I-A (16%) or I-X (7%) genetic signatures. Gliomas with I-A tumors were significantly younger than patients with I-CF tumors and patients with I-X tumors (mean ages 34, 44, and 54 years, respectively). Among all grade II – IV glioma patients, those whose tumors bore the I-A and I-CF signatures survived significantly longer than those with I-X tumors (median 51, 96, and 13 months, respectively), whereas in grade II glioma or grade II – III oligodendroglioma patients, no significant differences in median survival between the three groups could be observed. 

The prognostic impact of *ATRX* was corroborated in a second independent study, performed on a cohort of 133 malignant gliomas, treated within the German NOA-04 trial (NCT00717210) [11]. In this study, *IDH* mutations, MGMT promotor methylation, immunohistochemical loss of nuclear ATRX expression, which has been shown to correlate with *ATRX* mutations [3], and status of chromosome arms 1p and 19q were analyzed. Loss of nuclear ATRX expression was detected in 33% of all tumors and was significantly higher in anaplastic astrocytomas (AA, 45%) than in anaplastic oligoastrocytomas (AOA, 27%) and low in anaplastic oligodendrogliomas (AO, 10%). It occurred almost exclusively in tumors harboring *IDH* mutations (42/98 *IDH* mutated tumors). Nuclear ATRX loss and 1p/19q co-deletion were almost mutually exclusive. Furthermore, no association between MGMT promoter methylation and ATRX loss was observed. Similar to the study of Jiao et al, [4] patients with *IDH* mutation and ATRX loss were younger than patients with *IDH* mutation and ATRX expression, or *IDH* wild-type patients (mean age 35.7, 46.8, and 54.1 years, respectively). 

In a multivariate Cox regression model only ATRX loss and 1p/19q status were significantly associated with prognosis, whereas the histological subtype, which was significantly correlated with survival in univariate analysis, lost its significance. Based on these results, the authors classified the tumors into three groups 1) “molecular astrocytomas”: tumors with *IDH* mutation and without 1p/19q co-deletion, including mixed AOA with ATRX loss and AA with and without ATRX loss; 2) “molecular oligodendrogliomas”: tumors with *IDH* mutation and ATRX expression including AOA harboring 1p/19q co-deletion and AO with and without 1p/19q co-deletion; 3) “molecular glioblastomas”: tumors with *IDH* wild-type. Outcome analyses showed that the group of molecular oligodendrogliomas had the best prognosis, followed by the molecular group of astrocytomas, whereas the molecular glioblastoma group had the poorest outcome. Importantly, in the group of molecular astrocytoma, patients with ATRX loss had a significantly better outcome than patients without ATRX loss, thus providing evidence that the molecular profile helps to refine the prognosis in malignant glioma patients. 

In contrast to gliomas of adult patients, *ATRX* mutations are very rare in pediatric anaplastic gliomas and low-grade gliomas and have to date never been found in pilocytic astrocytomas [8, 9, 14]. 

### ATRX in neuropathological practice 

**ATRX expression**

**Figure 1.**
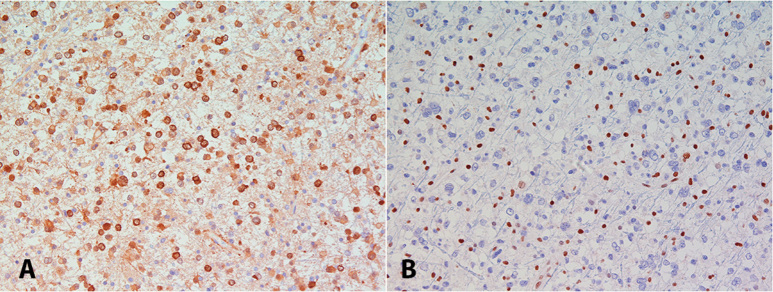
Diffuse astrocytoma with expression of mutant IDH1 protein (A) and loss of nuclear ATRX expression (B).

Loss of nuclear ATRX seems to be a good surrogate marker for *ATRX* mutations [3] and ATRX expression can – similarly to mutant IDH1 protein (Figure 1A) – be easily assessed in the routine neuropathological setting with a commercially available antibody (HPA001906, Sigma-Aldrich, St. Louis, MO, USA). Physiologically, ATRX protein is ubiquitously expressed in cell nuclei. Mutations in the *ATRX* gene result in a loss of nuclear protein expression in tumor cells, but retained expression in non-tumor cells (e.g., endothelial cells, pre-existing glial cells), which serve as a positive internal control (Figure 1B), analogously to the expression of INI1/SMARCB1 protein in atypical teratoid/rhabdoid tumors. Yet, interpretation of the immunohistochemical staining results might be difficult in diffusely infiltrating tumors with very low tumor cell content and areas with squeezing artifacts which are usually not well stained. 

**Diagnostic value of ATRX**

As *ATRX* mutations have been to date not detected in pilocytic astrocytomas, ATRX expression analysis might be helpful in diagnostically challenging small biopsies, especially with regard to the differential diagnosis of pilocytic versus diffuse astrocytoma. 

### Prognostic value of ATRX 

*ATRX* is a prognostic candidate biomarker in adult patients with malignant gliomas, where it might help to define a group of anaplastic astrocytomas with a better prognosis. The currently available literature suggests that anaplastic astrocytomas, oligodendrogliomas, and mixed oligoastrocytomas can be divided based on *IDH* and *ATRX* mutations and 1p/19q status in three prognostically different groups:

Tumors with *IDH* mutations, no *ATRX* mutation and 1p/19q co-deletion. These tumors are most frequently oligodendrogliomas and oligoastrocytomas and have the best prognosis.
Tumors with *IDH* mutations and *ATRX* mutation and without 1p/19q co-deletion. These tumors are most frequently astrocytomas or oligoastrocytomas and show an intermediate but significantly better prognosis than group 3 tumors.
Tumors without *IDH* mutation. These tumors have the poorest prognosis and seem to behave clinically like glioblastomas.

## Open questions

The impact of *ATRX* as prognostic marker in low-grade gliomas is to date still unclear.
A fraction of gliomas (e.g., *IDH*-mutated tumors with or without both *ATRX* mutation and 1p/19 co-deletion) cannot be classified according to the proposed algorithm.
The association between *ATRX* mutations and loss of nuclear expression should be confirmed in larger series.
As the histopathological diagnosis of glioma subtypes is prone to interobserver variability and molecular markers seem to improve the prediction of biological behavior, it will be a matter of discussion whether and how *IDH*, 1p/19q status, and *ATRX* can be used to complement and refine the diagnosis of glioma subtypes.

In conclusion, *ATRX* seems to be a promising candidate biomarker in gliomas, which could help to refine, in combination with *IDH* and 1p/19q status, the prognosis of patients with malignant gliomas. 

## Conflict of interest 

The authors declare no conflict of interest.
